# Primary Human Renal Proximal Tubular Epithelial Cells (pHRPTEpiCs): Shiga Toxin (Stx) Glycosphingolipid Receptors, Stx Susceptibility, and Interaction with Membrane Microdomains

**DOI:** 10.3390/toxins13080529

**Published:** 2021-07-28

**Authors:** Johanna Detzner, Anna-Lena Klein, Gottfried Pohlentz, Elisabeth Krojnewski, Hans-Ulrich Humpf, Alexander Mellmann, Helge Karch, Johannes Müthing

**Affiliations:** 1 Institute of Hygiene, University of Münster, D-48149 Münster, Germany; johanna.detzner@ukmuenster.de (J.D.); kleianna96@gmail.com (A.-L.K.); pohlentz@uni-muenster.de (G.P.); lilly.kroj@gmail.com (E.K.); Alexander.Mellmann@ukmuenster.de (A.M.); hkarch@uni-muenster.de (H.K.); 2 Institute of Food Chemistry, University of Münster, D-48149 Münster, Germany; humpf@uni-muenster.de

**Keywords:** detergent-resistant membranes, glycolipids, kidney epithelial cells, lipid rafts, Stx1a, Stx2a, surface acoustic wave

## Abstract

Tubular epithelial cells of the human kidney are considered as targets of Shiga toxins (Stxs) in the Stx-mediated pathogenesis of hemolytic–uremic syndrome (HUS) caused by Stx-releasing enterohemorrhagic *Escherichia coli* (EHEC). Analysis of Stx-binding glycosphingolipids (GSLs) of primary human renal proximal tubular epithelial cells (pHRPTEpiCs) yielded globotriaosylceramide (Gb3Cer) and globotetraosylceramide (Gb4Cer) with Cer (d18:1, C16:0), Cer (d18:1, C22:0), and Cer (d18:1, C24:1/C24:0) as the dominant lipoforms. Investigation of detergent-resistant membranes (DRMs) and nonDRMs, serving as equivalents for the liquid-ordered and liquid-disordered membrane phase, respectively, revealed the prevalence of Gb3Cer and Gb4Cer together with cholesterol and sphingomyelin in DRMs, suggesting lipid raft association. Stx1a and Stx2a exerted strong cellular damage with half-maximal cytotoxic doses (CD_50_) of 1.31 × 10^2^ pg/mL and 1.66 × 10^3^ pg/mL, respectively, indicating one order of magnitude higher cellular cytotoxicity of Stx1a. Surface acoustic wave (SAW) real-time interaction analysis using biosensor surfaces coated with DRM or nonDRM fractions gave stronger binding capability of Stx1a versus Stx2a that correlated with the lower cytotoxicity of Stx2a. Our study underlines the substantial role of proximal tubular epithelial cells of the human kidney being associated with the development of Stx-mediated HUS at least for Stx1a, while the impact of Stx2a remains somewhat ambiguous.

## 1. Introduction

Shiga toxins (Stxs) are powerful bacteriogenic AB_5_ toxins and the primary virulence factors of human–pathogenic enterohemorrhagic *Escherichia coli* (EHEC), which represent a sublineage of Stx-producing *Escherichia coli* (STEC) [1] with emerging public health challenges [2]. EHEC are responsible for bloody diarrhea and Stx-mediated extraintestinal complications such as life-threatening hemolytic–uremic syndrome (HUS) and neurological disturbances [3] exhibiting tremendous global outbreak potential [4,5,6]. HUS is the leading cause of acute kidney injury in children [7] comprising of thrombocytopenia, microangiopathic hemolytic anemia, and renal failure [8]. Ruminant animals are deemed to serve as a critical environmental reservoir of STEC [9,10]. The rapid detection of STEC at genetic and phenotypic level enables appropriate monitoring, assessment of the relative virulence of the strains, and treatment of STEC infections [11,12]. Protection can be provided either by inhibiting the binding of Stx toward the cell surface using therapeutics based on chemical analogs of the Stx receptor [13,14,15], interfering of small-molecule inhibitors with any of the subsequent steps upon retrograde trafficking that act at the endosome/Golgi interface required for the toxin’s intracellular destructive effects [16,17,18], or blocking of transcriptional and translational inhibitors that may be of value in treating EHEC infections [19]. Despite decades of work elucidating the mechanisms of Stx toxicity in sensible cells, no specific treatment exists for STEC-induced diseases, and recommended therapy today is mainly supportive [20,21]. Stx-specific therapeutics based on chemical analogs of the Gb3 oligosaccharide, although effective in vitro, have failed so far in vivo [15]. However, the monoclonal antibody eculizumab against the human complement C5 protein has proven effective in some cases and shown positive clinical improvement in severe STEC-HUS with progressive neurological involvement [22,23,24]. 

Stxs are bacterial type 2 ribosome-inactivating proteins (RIPs) and belong to the group of AB_5_ enterotoxins, which comprise a catalytic A chain with *N*-glycosidase activity and five identical B chains with binding specificity toward certain cell surface carbohydrate structures [25]. Similar to the heterodimeric plant type 2 RIPs ricin and viscumin with AB structure, Stxs catalyze the cleavage of an adenine residue of the universally conserved α-sarcin/ricin loop at the 28S rRNA of the eukaryotic 60S ribosomal subunit, resulting in irreversible disruption of the protein synthesis [26,27,28,29]. The B subunit of ricin binds to β-configurated galactose in distal position and that of viscumin (synonymous with mistletoe lectin I) to terminally β-configurated galactose occupied with an *N*-acetylneuraminic acid (Neu5Ac) in α2-6-linkage [30]. The α2-6-linked Neu5Ac is a common constituent of *N*-glycans of glycoproteins and glycosphingolipids (GSLs) of the neolacto-series exposed on the plasma membrane of human target cells where it is accessible for viscumin [31]. In contrast to ricin and viscumin, the B pentamer of the human–pathogenic Stx subtypes Stx1a and Stx2a preferably recognizes the terminally α1-4-linked galactose of the GSL globotriaosylceramide (Gb3Cer, Galα1-4Galβ1-4Glcβ1-1Cer), whereas the swine-pathogenic Stx2e subtype binds globotetraosylceramide (Gb4Cer, GalNAcβ1-3Galα1-4Galβ1-4Glcβ1-1Cer) [32] just as well when compared to Gb3Cer [33]. The subsequently endocytosed Stx-GSL complex has been reported to follow various retrograde routes via early endosomes through the Golgi network to the endoplasmic reticulum, where the A1 fragment of the A subunit exerts its cytotoxic effect [34,35,36,37]. In addition, Stx (similarly to a number of other RIPs) is known to efficiently depurinate nuclear DNA due to its polynucleotide:adenosine glycosidase activity [27,38,39]. Moreover, it turned out in the past two decades that Stxs are multi-functional proteins and capable of modulating a wealth of vital cellular functions at the molecular level [1]. Experimentally verified manifold modes of action of Stx beyond its canonical ribotoxic activity include the activation of multiple cell stress signaling pathways, which may result in apoptosis, autophagy, or stimulation of the innate immune response [40,41]. Remarkably enough, an ex vivo study of human erythropoiesis has shown a cell injury effect of Stx toward human hematopoietic stem/progenitor cells, suggesting the involvement of Stx in the manifestation of anemia in patients suffering from EHEC infections [42,43]. 

The high and less efficient Stx-binding GSLs Gb3Cer and Gb4Cer, respectively, are primarily expressed by microvascular endothelial cells of renal glomeruli and the human brain [44,45,46,47], which are considered as the chief targets of human-pathogenic Stx1a and Stx2a [1,15,48,49]. On the other side, immortal kidney epithelial cell lines derived from human tubule epithelium are widely used as in vitro models of Stx-caused damage of the human kidney. Examples are the cell lines HK-2 [50,51,52,53] and ACHN [52,53], which are sensitive toward Stx. The main Stx receptor Gb3Cer has been detected in HK-2 and ACHN [52,54], and the exact structures of the various Gb3Cer lipoforms of ACHN cells recognized by Stx have been recently described [17]. Importantly, primary human renal cortical epithelial cells do contain the Stx receptor GSL Gb3Cer and are susceptible toward the cytotoxic effects of Stxs as well [55,56,57]. Responsiveness toward Stx has been reported for primary human renal tubular epithelial cells [58,59,60,61,62]. These results suggest the involvement of kidney epithelial cells, beside the endothelial cells of the kidney microvasculature, in EHEC-HUS supported by an appropriate mouse model, which has shown the direct contribution of tubular damage to Stx-mediated kidney failure [63,64]. Although the interaction of Stx with human intestinal epithelial cell lines has been shown, the human intestinal epithelium seems to lack the Stx receptor Gb3Cer, and it remains unknown how Stx crosses the intestinal barrier and gains access to the systemic circulation [65,66]. Once transferred into the human bloodstream, Stx-loaded neutrophils and/or vesicle-associated Stx may play a functional role in the development of HUS in the process of delivering the toxin to renal microvascular endothelial cells [67,68,69,70].

We have recently characterized the major and minor Stx-binding Gb3Cer and Gb4Cer species of primary human renal cortical epithelial cells (pHRCEpiCs), scrutinized their distribution to detergent-resistant membranes (DRMs) (used as lipid raft equivalents), determined the GSLs’ environmental phospholipids in the membrane microdomains, and analyzed the cellular sensitivity toward the Stx subtypes Stx1a and Stx2a [71]. Here, we report on a further kidney epithelial cell type, possibly being involved in the manifestation of EHEC-HUS, providing a comprehensive investigation on primary human renal proximal tubular epithelial cells (pHRPTEpiCs). Beyond the fine characterization of the Stx receptors and flanking phospholipids in microdomains as well as determining the Stx sensitivity as recently described by us for pHRCEpiCs [71], we additionally applied in this study the surface acoustic wave (SAW) technology showing real-time interaction of Stx1a and Stx2a with DRM and nonDRM preparations of pHRPTEpiCs.

## 2. Results

As a follow-on project with regard to our recently published data on Stx-binding GSLs of pHRCEpiCs and their susceptibility toward the Stx1a and Stx2a subtypes [71], we continue in this article with a comprehensive and detailed investigation on the characteristics of pure pHRPTEpiCs, which line the proximal renal tubules. Two independent biological replicates, replicate 1 (R1) and replicate 2 (R2), were prepared from pHRPTEpiCs derived from early passages. Due to limited quantities of cell material from labor-intensive cultures of primary epithelial cells, a complete set of experiments of each of the two replicates was not feasible. Thus, some analyses were performed with material of replicate 1 and some other using material from replicate 2 as outlined in the following sections.

### 2.1. Detection of Stx1a- and Stx2a-Binding GSLs of pHRPTEpiCs

**Figure 1:**
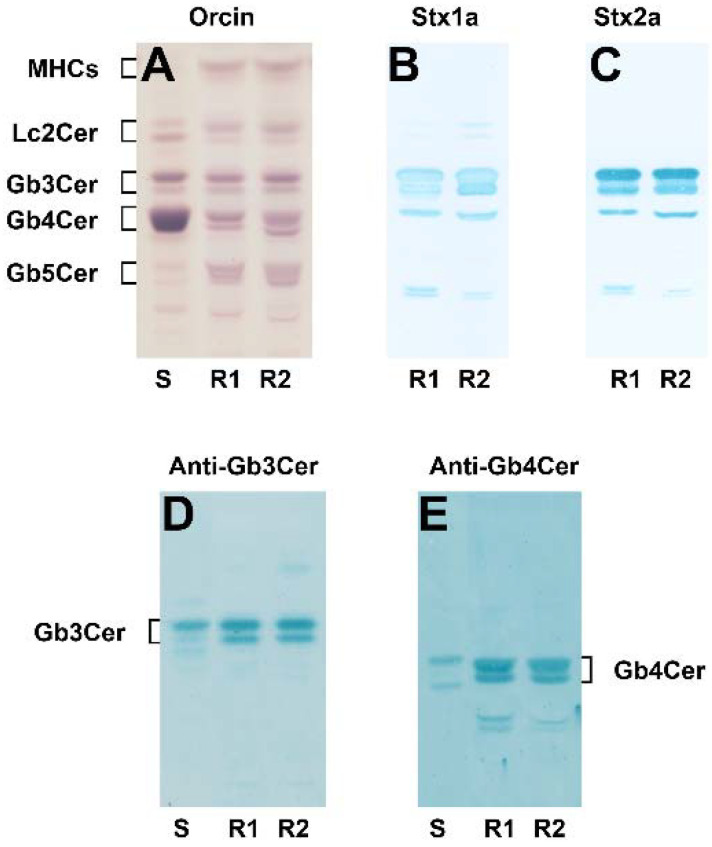
Orcinol stain (**A**) and TLC overlay assays of the neutral GSL preparation obtained from pHRPTEpiCs using Stx1a (**B**) and Stx2a (**C**) as well as anti-Gb3Cer (**D**) and anti-Gb4Cer (**E**) antibody. The employed GSL quantities for TLC separation were equivalent to 5 × 10^6^ cells (A, orcinol stain), 6 × 10^5^ cells (**B**,**C**, Stx1a and Stx2a, respectively) and 2 × 10^6^ cells using the anti-Gb3Cer (**D**) and the anti-Gb4Cer antibody (**E**), respectively. S, 20 µg of a standard GSL mixture prepared from human erythrocytes (**A**), and 2 µg and 0.2 µg for the anti-Gb3Cer (**D**) and the anti-Gb4Cer (**E**) TLC immunodetection, respectively; R1, replicate 1; R2, replicate 2; MHCs, monohexosylceramides; Lc2Cer, lactosylceramide. Cells of the fifth passage were used for GSL isolation.

The orcinol stain of the neutral GSLs prepared from replicate (R1) and replicate 2 (R2) of pHRPTEpiCs and the corresponding thin-layer chromatography (TLC) overlay assays using Stx1a and Stx2a as well as Gb3Cer- and Gb4Cer-specific antibodies are shown in Figure 1. Lipid isolation was performed using cells of the fifth passage. Cells of higher passages (>passage 8) start with exhibiting signs of senescence and dedifferentiation as shown in Figure S1 in the Supplementary Materials and should be expelled from analysis. Compared to the orcinol stain (Figure 1A), the Stx1a and Stx2a TLC overlay assays revealed strong and identical binding toward the double band of Gb3Cer, as demonstrated in Figure 1B,C, respectively, whereas only a weak interaction could be observed in case of Gb4Cer with a slightly positive upper band of the Gb4Cer doublet. TLC immunodetection with the GSL-specific antibodies confirmed proposed structures of Gb3Cer (Figure 1D) and Gb4Cer (Figure 1E) as the Stx-binding GSLs.

### 2.2. Mass Spectrometric Characterization of the Neutral GSL Fraction of pHRPTEpiCs

**Figure 2:**
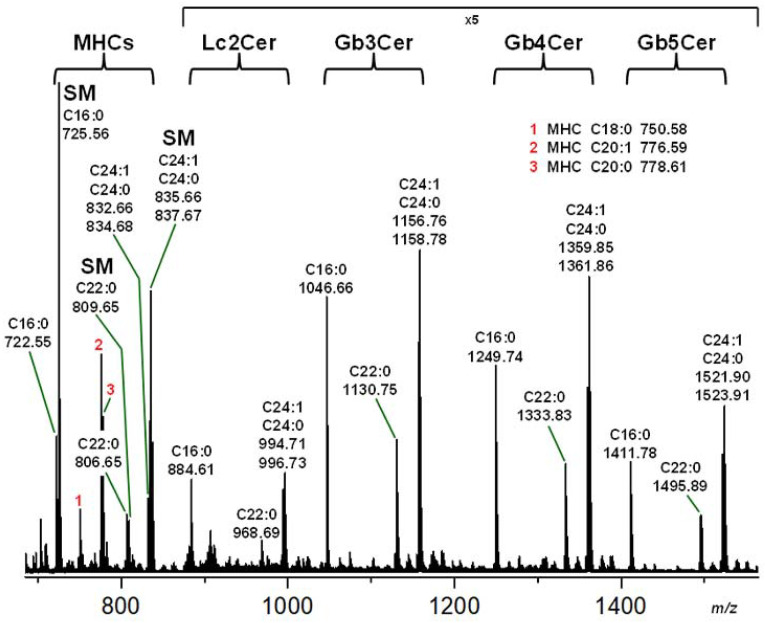
MS^1^ spectrum of the sphingolipid preparation including neutral GSLs and sphingomyelins obtained from pHRPTEpiCs. The sphingolipids were isolated from cells of the 5th passage of biological replicate 2 (R2). The orcinol stain of the TLC-separated GSLs is shown in Figure 1A accompanied by the Stx1a, Stx2a, anti-Gb3Cer, and anti-Gb4Cer TLC overlay assays depicted in Figure 1B–E. Mono-, di-, tri-, tetra-, and pentahexosylceramides were identified as monohexosylceramides (MHCs), Lc2Cer, Gb3Cer, Gb4Cer, and Gb5Cer, respectively. The sphingolipids were detected as monosodiated [M+Na]^+^ species operating in the positive ion mode and are displayed in Table 1. Structural proofs were performed by CID experiments, and examples of MS^2^ spectra are given for Gb3Cer (d18:1, C22:0), Gb4Cer (d18:1, C16:0), and proposed Gb5Cer (d18:1, C22:0) in Figures S2–S4, respectively, in the Supplementary Materials.

The structural characterization of the sphingolipids detectable in the total neutral GSL preparation of replicate 2 (see orcinol stain in Figure 1A) by means of electrospray ionization mass spectrometry (ESI-MS) revealed a collection of mono- to pentahexosylceramides including sphingomyelin (SM) as the main compounds proposed from the MS^1^ spectrum shown in Figure 2. Monohexosylceramides (MHCs) and lactosylceramide (Lc2Cer), representing the precursor molecules of higher glycosylated ceramides, accompanied by the potential Stx1a and Stx2a receptors Gb3Cer and Gb4Cer together with globopentaosylceramide (Gb5Cer, Galβ1-3GalNAcβ1-3Galα1-4Galβ1-4Glcβ1-1Cer) could be identified. SM and GSLs appear as monsodiated [M+Na]^+^ ions, indicating variable lipoforms with a constant sphingosine (d18:1) moiety linked to a fatty acid that varies in chain length from C16 to C24 as denoted in the MS^1^ spectrum (Figure 2). The major ceramide cores found are those with C16:0, C22:0, and C24:1/C24:0 fatty acids. Structural proof of the proposed structures was performed by collision-induced dissociation (CID) experiments. MS^2^ spectra of Gb3Cer (d18:1, C22:0), Gb4Cer (d18:1, C16:0), and Gb5Cer (d18:1, C22:0), together with the corresponding fragmentation schemes, are exemplarily shown in Figures S2–S4 in the Supplementary Materials, respectively. The identified main GSL and SM lipoforms are listed in Table 1. 

### 2.3. Distribution of Gb3Cer, Gb4Cer, and Cholesterol among DRM and nonDRM Fractions of pHRPTEpiCs

**Figure 3:**
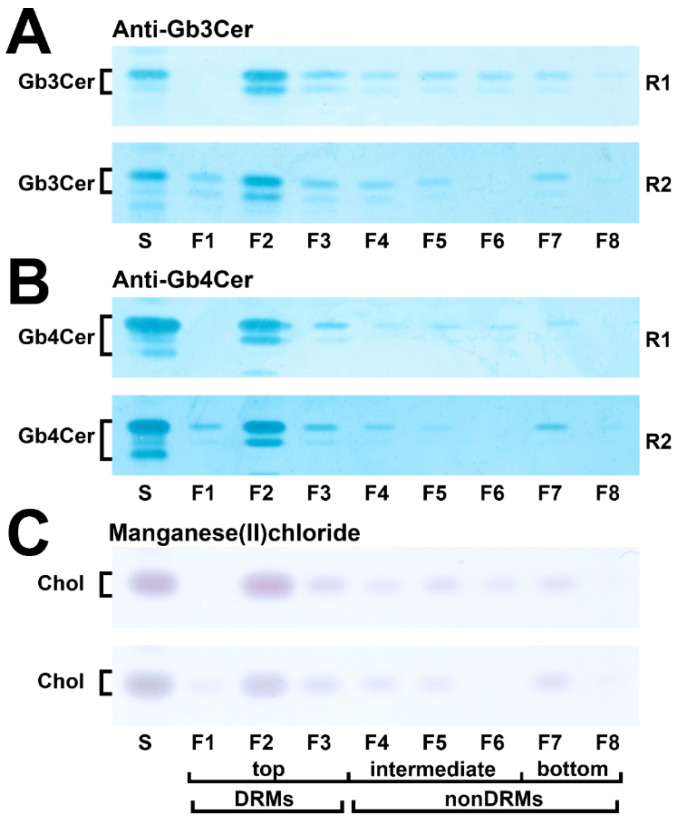
Occurrence of Gb3Cer (**A**), Gb4Cer (**B**), and cholesterol (**C**) in sucrose gradient fractions F1 to F8 of the two biological replicates obtained from pHRPTEpiCs. TLC-separated Gb3Cer and Gb4Cer as well as cholesterol (Chol) were detected in the respective gradient fractions of replicate 1 (R1) and replicate 2 (R2) by means of the anti-Gb3Cer and anti-Gb4Cer TLC overlay assay, respectively, and cholesterol bands with manganese(II)chloride. Each fractionation corresponds to 5 × 10^6^ cells. The GSL standard mixture of neutral GSLs from human erythrocytes (S) was equivalent to 2 µg and 0.2 µg for the detection of Gb3Cer and Gb4Cer, respectively; cholesterol standard (S), 1 µg. DRMs, detergent-resistant membranes.

The possible association of Gb3Cer, Gb4Cer, and cholesterol with the liquid-ordered and liquid-disordered membrane phase was probed using DRM (top, F1–F3) and nonDRM (intermediate F4–F6 and bottom F7–F8) fractions. The results are shown in Figure 3. The TLC immunodetection of Gb3Cer of both replicates (R1 and R2) indicates very similar distribution with preferential occurrence of Gb3Cer in the DRM fractions F1–F3 (Figure 3A). The same holds true for the distribution of Gb4Cer, which indicates also preferred occurrence of Gb4Cer in the DRMs (Figure 3B). The dominance of cholesterol in the canonical DRM fraction F2 (Figure 3C) correlates with the distribution of the two GSLs.

**Figure 4:**
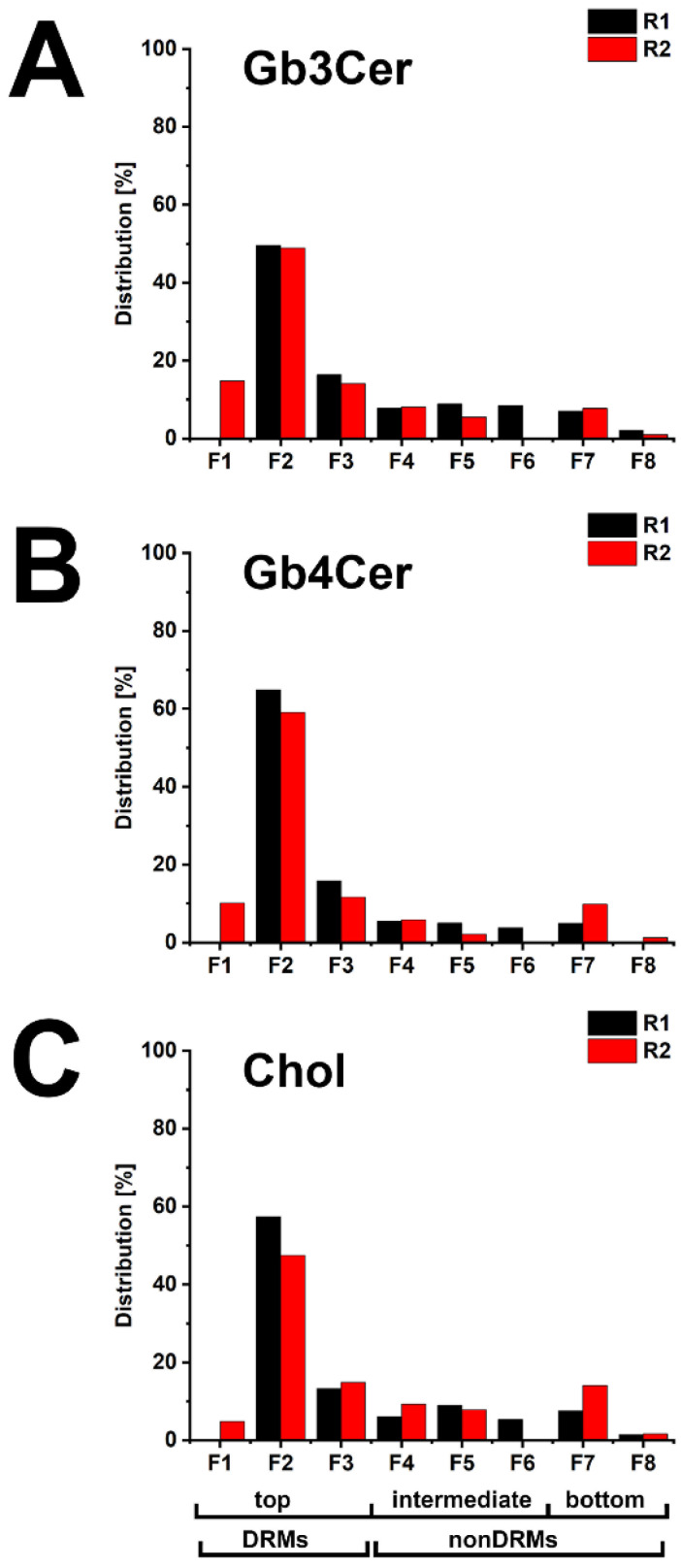
Distribution of Gb3Cer (**A**), Gb4Cer (**B**), and cholesterol (**C**) to sucrose gradient fractions F1 to F8 of the two biological replicates R1 and R2 obtained from pHRPTEpiCs. The TLC immunopositive Gb3Cer and Gb4Cer and the cholesterol bands shown in Figure 3 were densitometrically quantified, and each fractionation was normalized to 100% as displayed in the bar diagrams for Figure 3.

More precisely, the bar diagrams depicted in Figure 4 provide a detailed portrayal of the distribution of Gb3Cer, Gb4Cer, and cholesterol to the DRM (top) and nonDRM (intermediate and bottom) fractions showing a high degree of conformity of the two replicates (for a list of determined relative values, refer to Table S1 in the Supplementary Materials). The average value of the summed F1–F3 DRM fractions of the two replicates amounted to 71.9% for Gb3Cer, 80.9% for Gb4Cer, and 69.0% for cholesterol, suggesting the possible association of these membrane compounds with lipid rafts.

### 2.4. Lipoform Variability of Gb4Cer in DRM and nonDRM Fractions

**Figure 5:**
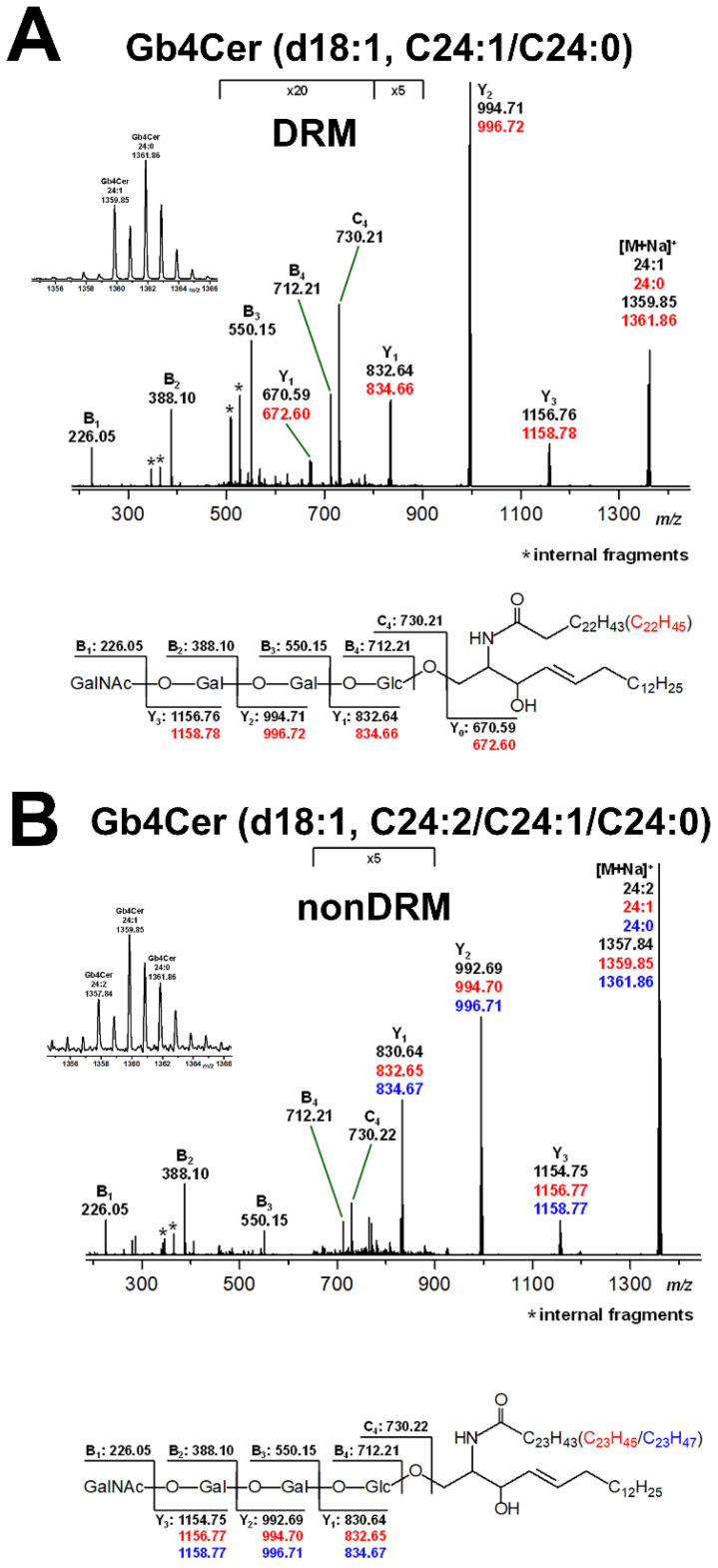
MS^2^ spectra and explanatory fragmentation schemes of Gb4Cer (d18:1, C24:1/C24:0) of DRM fraction F2 (**A**) and Gb4Cer (d18:1, C24:2/C24:1/C24:0) of nonDRM fraction F7 (**B**) obtained from replicate 2 of pHRPTEpiCs. The expanded *m/z* range of the [M+Na^+^] precursor ions at *m/z* 1359.85/1361.86 (**A**, insert) and those at *m/z* 1357.84/1359.85/1361.86 shown (**B**, insert) illustrate the different Gb4Cer lipoforms harboring a C24:2, C24:1, and C24:0 fatty acid. The MS^2^ spectra demonstrate, together with the corresponding fragmentation schemes, the proof of structure of the MS^1^-deduced proposed Gb4Cer species, each carrying a ceramide portion composed of a uniform sphingosine (d18:1) moiety and a fatty acid that varies in chain saturation as indicated. For further details of the gradient fractions, refer to the captions of Figure 3 and Figure 4.

We were successful in performing MS^2^ analysis of the Gb4Cer lipoforms in the DRM fraction F2 and the nonDRM fraction F7 of replicate 2 (see Figure 3B) carrying variable C24 fatty acids, as shown in Figure 5A,B, respectively. The MS^2^ spectrum of Gb4Cer (d18:1, C24:1/C24:0) from DRM fraction F2 (Figure 5A) indicated a slight preference of Gb4Cer with saturated C24:0 over the counterpart with monounsaturated C24:1 fatty. By contrast, the parallel analysis of the DRM fraction F7 (Figure 5B) revealed the presence of Gb4Cer (d18:1, C24:2/C24:1/C24:0) with the prevalence of Gb4Cer with monounsaturated C24:1. Importantly, the Gb4Cer species with two-fold unsaturated C24:2 fatty is unique for the nonDRM fraction F7 and has been detected neither in the total GSL fraction (see Figure 2) nor in the DRM fraction F2 (see Figure 5A). 

### 2.5. Mass Spectrometric Characterization of Phospholipids in DRM and nonDRM Fractions of pHRPTEpiCs

**Figure 6:**
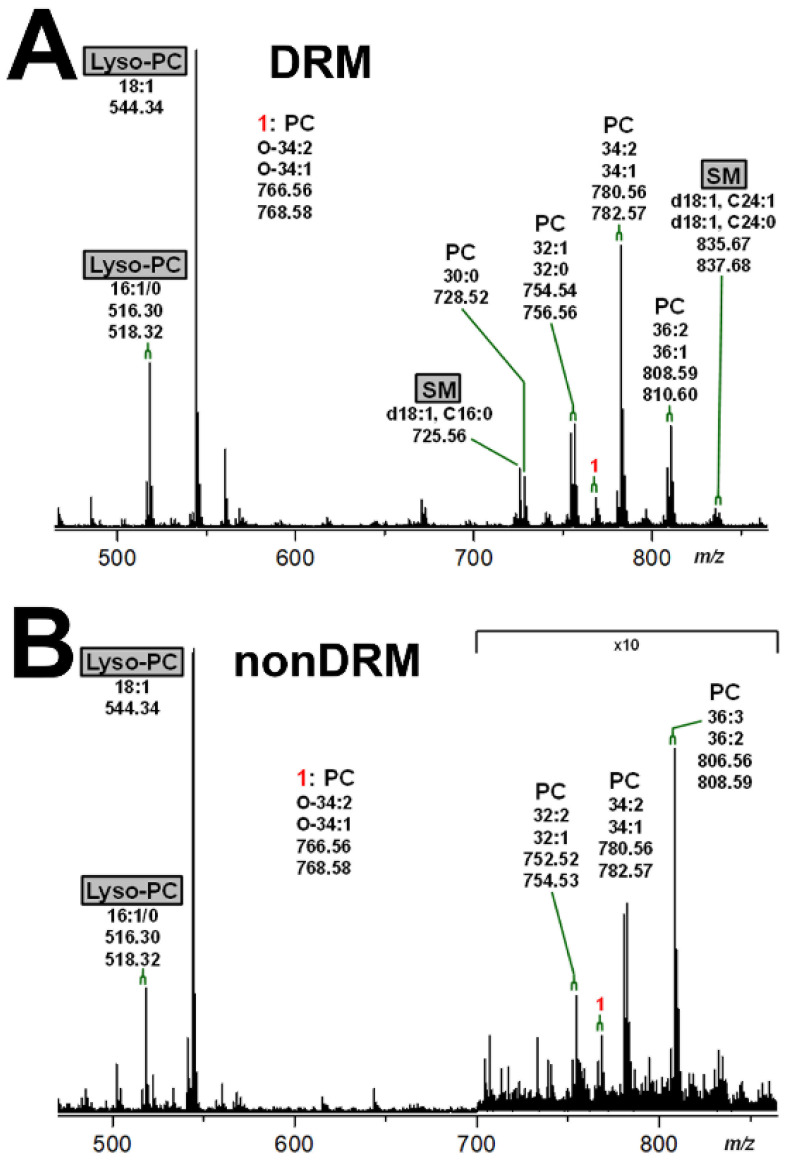
MS^1^ spectra of phospholipids of DRM fraction F2 (**A**) and nonDRM fraction F7 (**B**) obtained from replicate 2 of pHRPTEpiCs. The spectra were recorded in the positive ion mode yielding monosodiated [M+Na]^+^ species, which could be assigned to the phospholipids indicated. SM species (gray boxes) highlight this characteristic marker of the liquid-ordered membrane phase, whereas lyso-PC species (gray boxes) appear in both the liquid-ordered (F2) and the liquid-disordered membrane phase (F7). PC, phosphatidylcholine; SM, sphingomyelin.

The mass spectrometric specification of the glycerophospholipids and SM in DRM fraction F2 and nonDRM fraction F7 prepared from replicate 2 of pHRPTEpiCs is displayed in Figure 6. The PC (34:2/34:1) lipoform was predominant in DRM fraction F2, which was accompanied by less abundant PC (30:0), PC (32:1/32:0), and PC (36:2/36:1) (Figure 6A). The PC variants were flanked by small signals that could be assigned to SM (d18:1, C16:0) and SM (d18:1, C24:1/C24:0). The appearance of SM was restricted to the DRM fraction, suggesting this membrane lipid as a specific marker of the liquid-ordered membrane phase. Importantly, monotailed lyso-PC (18:1), along with the less abundant lyso-PC (16:1/0), exhibited the strongest signal in the spectrum. The group of PC lipoforms in the nonDRM fraction 7 consisted of PC (36:3/36:2), PC (34:2/34:1), and PC (32:2/32:1) (Figure 6B). Importantly, PC lipoforms with saturated acyl chains and SM were undetectable, while three-fold unsaturated PC (36:3) was detected as a unique PC variant. The same lyso-PC species apparent in the DRM fraction F2 were detected as high abundant phospholipid species in nonDRM F7. Taking the 10-fold amplification of the signal intensities of the PC species in the *m/z* range between 700 and 860 in the spectrum into consideration (Figure 6B), it becomes obvious that the signals of lyso-PC (18:1) and lyso-PC (16:1/0) dominated over those of the PC molecules in the nonDRM fraction F7, although different ionizability cannot be excluded, which might have an effect on the signal intensities of detected phospholipids.

Collectively, the GSLs Gb3Cer and Gb4Cer as well as cholesterol and SM distributed among the gradient fractions with high preference to the DRM fractions, suggesting their possible association with lipid rafts. Furthermore, a shift to an increased degree of unsaturation of the lipid anchors of the GSLs and PC was found characteristic for the nonDRM fraction F7 equating to the liquid-disordered membrane phase. 

### 2.6. Stx1a- and Stx2a-Effected Cellular Injury of pHRPTEpiCs

**Figure 7:**
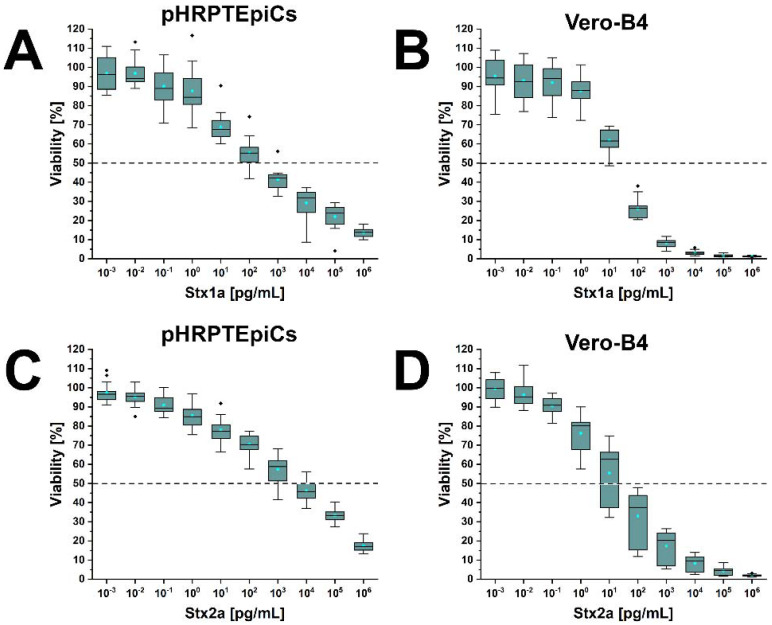
Cytotoxic response of pHRPTEpiCs (**A**,**C**) and the Vero-B4 reference cell line (**B**,**D**) to exposure of increasing concentrations of Stx1a and Stx2a. Cytotoxicity was quantified by means of the crystal violet assay showing the absorption readings of Stx1a- and Stx2a-challenged cells as box plot charts. Measured values are depicted as percentages in relation to 100% viability of untreated parallel cell cultures. Approaches were performed as 6-fold measurements of three independent cell culture replicates (one replicate of passage 4 and two replicates of passage 5).

Figure 7 shows the course of the survival rates of pHRPTEpiCs upon exposure to increasing concentrations of Stx1a (Figure 7A) and Stx2a (Figure 7C) compared to Stx1a- and Stx2a-treated Vero-B4 reference epithelial cells (Figure 7B,D, respectively). A significant initial sensitivity of pHRPTEpiCs toward Stx1a was recognized at a toxin concentration of 10^0^ pg/mL that affected a reduced cell viability of 92.1 ± 10.6% as shown in the box plot (Figure 7A). The concentration-dependent gradual decrease of cell viability continued down to 12.8 ± 1.9% survival upon treatment of the cells with 10^6^ pg/mL (equivalent to 1 µg/mL) of Stx1a. The 50% cytotoxic dose (CD_50_) of Stx1a was 1.31 × 10^2^ pg/mL for pHRPTEpiCs. The corresponding parameter for Vero-B4 cells was 1.33 × 10^1^ pg/mL, indicating one order of magnitude (factor 9.9) higher susceptibility toward Stx1a (Figure 7B). A more efficient cell killing rate was observed in case of Vero-B4 cells, resulting in an almost complete killing rate of the cell cultures at 10^5^ and 10^6^ pg/mL of Stx1a (1.8 ± 0.4% and 1.3 ± 0.3% survival, respectively) (Figure 7B).

A progressive increase in cellular damage upon exposure of pHRPTEpiCs toward Stx2a (Figure 7C) was found similar to that of Stx1a. A relevant toxin-mediated decrease in viability started at an Stx2a concentration of 10^−1^ pg/mL (90.9 ± 5.6% viability) and continuously rose to a final cell survival of 18.3 ± 2.8% applying 10^6^ pg/mL (equivalent to 1 µg/mL) of Stx2a. The CD_50_ of Stx2a for pHRPTEpiCs amounted to 1.66 × 10^3^ pg/mL, while the counterpart value for Vero-B4 was 1.25 × 10^1^ pg/mL corresponding to a more than two orders of magnitude (factor 132.8) higher sensitivity determined for Vero-B4 cells (Figure 7D). An almost entire killing rate was obtained with Stx2a at 10^5^ and 10^6^ pg/mL (5.5 ± 1.3% and 2.2 ± 0.6% survival, respectively) (Figure 7D). 

In sum, Stx1a exhibited a more than one order of magnitude (factor 12.7) higher cytotoxic activity against pHRPTEpiCs (CD_50_ Stx1a of 1.31 × 10^2^ versus CD_50_ Stx2a of 1.66 × 10^3^ pg/mL) based on the comparison of the 50% cytotoxic doses.

### 2.7. Real-Time Interaction Analysis of Stx1a and Stx2a with DRM and nonDRM Fractions of pHRPTEpiCs 

**Figure 8:**
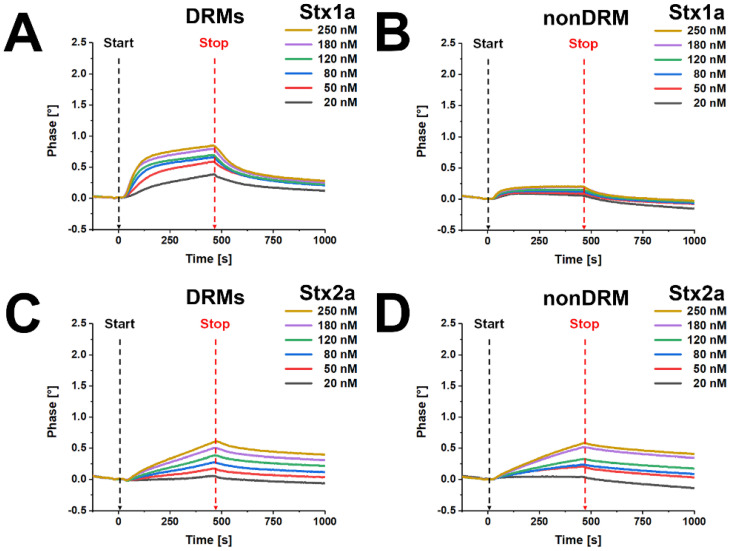
SAW real-time interaction sensorgrams gained for binding of Stx1a and Stx2a toward DRM (**A**,**C**) and nonDRM fractions (**B**,**D**) prepared from replicate 2 of pHRPTEpiCs. The biosensor surface was coated with pooled DRMs (F1 to F3) (**A**,**C**) or nonDRM fraction F7 (**B**,**D**), and the interaction of Stx1a and Stx2a was portrayed for the representative channel 3 of the biochips as association and dissociation curves using increasing toxin concentrations as indicated. Start: begin of toxin exposure; stop: end of toxin exposure.

The binding curves obtained with Stx1a and Stx2a for DRM and nonDRM fractions of replicate 2 are displayed in Figure 8. The application of increasing concentrations of Stx1a to the DRM-coated biochip resulted in characteristic association and dissociation curves (Figure 8A). A steep increase of attachment occurred immediately after starting the injection, leading to a plateau-like setting at approximately 125 s using ≥ 80 nM toxin concentrations, whereas only extremely weak and de facto irrelevant attachment was determined using a nonDRM-coated biochip (Figure 8B). However, a recognizable adhesion was detected for Stx1a toward the nonDRM preparation of replicate 1, as shown in Figure S5 in the Supplementary Materials. 

The real-time interaction of Stx2a with DRM and nonDRM fractions was rather indifferent, showing an almost identical but low strength in binding intensities with regard to the DRMs (Figure 8C) and the nonDRM preparation (Figure 8D). Data determined for Stx2a using DRMs from replicate 1 were comparable to those of replicate 2, as demonstrated in Figure S6 in the Supplementary Materials. 

**Figure 9:**
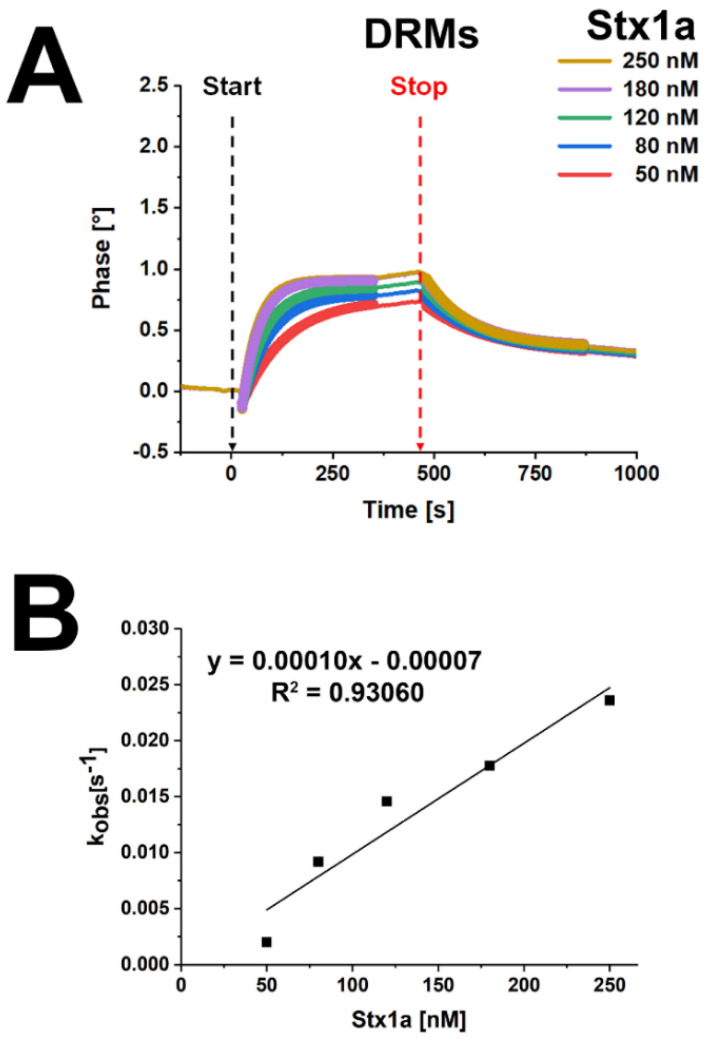
SAW real-time interaction sensorgram of Stx1a with DRM fractions prepared from replicate 1 of pHRPTEpiCs (**A**) and function graph according to the equation *k_obs_* = *k_ass_* × *c* + *k_diss_*, yielding the calculated dissociation constant K_D_ of Stx1a (**B**). The interactions between increasing concentrations of Stx1a and pooled DRMs (F2 and F3) (see Figure 8) are portrayed as association curves (ascending bold lines, left side of the panel) and dissociation curves (descending bold lines, right side of the panel). The calculated rounded rate constants *k_ass_* and *k_diss_* and the equilibrium dissociation constant *K_D_* of the real-time interaction analysis using five-channel measurements of DRMs are summarized in Table S1 in the Supplementary Materials. Start: begin of Stx injection; stop, end of Stx exposure.

A more precise binding analysis of Stx1a with DRMs from replicate 1 could be performed by calculation of the association *k_ass_* and dissociation *k_diss_* rate constants and the equilibrium dissociation constant *K_D_*, as shown in Figure 9. The individual and averaged *k_ass_*, *k_diss_*, and *K_D_* values are listed in Table S2 in the Supplementary Materials. The calculated *K_D_* mean value was 79.5 ± 5.7 nM, indicating high-affinity binding of Stx1a underlined by the function graph according to the equation *k_obs_* = *k_ass_* × *c* + *k_diss_* (Figure 9B). 

In summary, from our SAW interaction analysis, we can conclude a high binding potential of Stx1a toward DRMs, which contained the highest relative amounts of Gb3Cer and Gb4Cer among the gradient fractions, whereas a low binding capacity was observed for Stx2a toward DRMs. Furthermore, the results suggest a preferential binding of Stx1a to microdomains of the liquid-ordered membrane phase, which renders Stx1a different from Stx2a, although both toxins exhibited a comparable cytotoxic activity toward pHRPTEpiCs ranging in the same order of magnitude.

## 3. Discussion

Since the exact structures of Stx-recognized GSLs of primary epithelial cells derived from human kidney tubules and their membrane distribution as well as embedding membrane lipids have not been determined to this day, we scrutinized these parameters of primary human renal epithelial cells isolated from the proximal tubuli, abbreviated with pHRPTEpiCs, in this study. DRM and nonDRM fractions served as equivalents of the liquid-ordered and the liquid-disordered membrane phase, respectively.

The structural characterization of the globo-series GSLs Gb3Cer, Gb4Cer, and Gb5Cer and the accompanying precursor GSLs (MHCs and Lc2Cer) from pHRPTEpiCs revealed a variety of lipoforms. Gb3Cer was identified as highly efficient and Gb4Cer as a less efficient receptor for Stx1a and Stx2a, while Gb5Cer is not an attachment structure neither for Stx1a nor for Stx2a. The ceramide lipoforms Cer (d18:1, C16:0), Cer (d18:1, C22:0), and Cer (d18:1, C24:1/C24:0), carrying constant sphingosine (d18:1) and a fatty acid that varies in chain length, dominated in the neutral GSL preparation of pHRPTEpiCs. The biological relevance and in particular the biological function of this stable feature of endothelial and epithelial cells remain largely unknown. However, interdigitating sphingolipids of the lipid outer leaflet of the plasma membrane with long-chain C24 fatty acids may interact with phosphatidylserine (36:1) in the inner leaflet, a process, which is known as “hand-shaking” [72,73]. The mentioned Gb3Cer and Gb4Cer structures harboring Cer (d18:1, C16:0), Cer (d18:1, C22:0), and Cer (d18:1, C24:1/C24:0) have been previously identified as major neutral GSLs of human endothelial cells derived from two different vascular beds, videlicet human brain (pHBMECs) [74] and human kidney (pHRGECs) [75], as recently summarized in a review by Legros et al. [47]. Moreover, the same dominance of the mentioned Gb3Cer and Gb4Cer lipoforms was reported for human epithelial cells of the cortex (pHRCEpiCs) [71] and the proximal tubules (pHRPTEpiCs), as shown in this study. These findings suggest the ubiquitous presence of Gb3Cer and Gb4Cer in endothelial cells of the human brain and kidney as well as different types of human renal epithelial cells. Interestingly, due to its lack in human endothelial cells [47], Gb5Cer can be considered in this stage of research as a characteristic feature or marker of human kidney epithelial cells being present in pHRCEpiCs [71] and pHRPTEpiCs as described in this study, although the biological relevance remains obscure yet. However, in addition to these two types of kidney epithelial cells, Gb5Cer has been detected in GSL preparations of Vero cells representing kidney epithelial cells of the African green monkey [76] and thus can be postulated more generally as a marker of primate epithelial cells. However, this hypothesis requires further verification at this stage of research by detailed GSL analyses of epithelial cells from various primate populations. Interestingly, a “similar” pentahexosylceramide, namely the Forssman GSL with GalNAcα1-3Gb4Cer structure, was detected—in addition to Gb3Cer and Gb4Cer—in the canine kidney epithelial cell line MDCK II [77], exhibiting a different distal sugar moiety but with an identical Gb4Cer core when compared to Gb5Cer, which has the Galβ1-3Gb4Cer structure. With respect to the Stx binding specificities, the Forssman GSL and Gb5Cer are recognized by the swine-pathogenic Stx2e subtype, whereas Stx1a and Stx2a do not bind to these two pentahexosylceramides.

The prevalence of Gb3Cer and Gb4Cer carrying saturated acyl chains in their ceramide moieties has been located in DRMs obtained from primary human endothelial cells of the brain (pHBMECs) and primary human epithelial cells of the kidney cortex (pHRCEpiCs) [71,78] as well as for primary human epithelial cells of renal proximal tubules (pHRPTEpiCs), as shown in this study. This feature of Gb3Cer and Gb4Cer suggests their association with lipid rafts, which is an expectation that was further corroborated by the enrichment of cholesterol (“membrane glue”) and SM, the two canonical lipid raft markers, in the DRMs renowned as equivalents of the liquid-ordered membrane phase. Beyond that, the enrichment of monounsaturated Gb3Cer and/or Gb4Cer lipoforms, i.e., monounsaturated fatty acid linked to the sphingoid base in the ceramide portion, has been recognized in nonDRM fractions obtained from pHBMECs and pHRCEpiCs [71,78] as well as for pHRPTEpiCs, as shown in this study. Moreover, Gb3Cer and/or Gb4Cer species with two-fold unsaturated C24:2 fatty acid have been previously shown to represent unique constituents of nonDRMs derived from pHBMECs and pHRCEpiCs [71,78]. The same distribution was found for pHRPTEpiCs in this study, whereby nonDRMs are considered as equivalents of the liquid-disordered membrane phase. In line with this shift to a higher degree of unsaturation of the lipid anchor of GSLs, the lipoforms of PC, the prevalent phospholipid in DRM and nonDRM fractions, exhibited the same trend to an increasing number of double-bonds in the respective acyl chains of the PC lipoforms occurring in nonDRMs of pHBMECs [74] and pHRGECs [75]. The same switch to a higher extent of unsaturated PC species was found for pHRCEpiCs [71] and pHRPTEpiCs, as demonstrated in this study. 

The cytotoxic activity of Stx toward cultured primary human tubular epithelia cells of the human kidney has been previously described in a number of reports [56,58,59,60,61,62,79,80,81]. Although primary cells require higher effort regarding cell cultivation due to their limited number of cell divisions throughout in vitro propagation, these natural cells are closer to the original tissue and correspond much better to the in vivo situation than immortal cell lines, which have been mostly isolated from tumor tissues. The 50% cytotoxic dose (CD_50_) of Stx1a deduced from the course of the survival rates was 1.31 × 10^2^ pg/mL for pHRPTEpiCs as determined in this study. The CD_50_ of Stx2a for pHRPTEpiCs amounted to 1.66 × 10^3^ pg/mL. Thus, Stx1a exerted a more than one order of magnitude (factor 12.7) higher cytotoxic activity against pHRPTEpiCs based on the comparison of the 50% cytotoxic doses. Early investigations have shown a high cytotoxic effect of Stx1(a) toward human proximal tubule cells equal to that seen for Vero cells [79] and also for Stx2(a)-exposed tubular epithelial cells [58]. More precisely, the treatment of human proximal tubular epithelial cells with verocytotoxin 1 (=Stx1a) induced primary signs of apoptosis upon toxin application at 100 pM (=6.9 pg/mL) [59]. In our cytotoxicity assays, the initiation of Stx1a-mediated cell damage occurred in a concentration range of Stx1a between 1 and 10 pg/mL of Stx1a (see Figure 9) being in perfect compliance with the data of Kodama et al. [59]. Similar results for this human epithelial cell type were obtained for Stx1(a)-mediated half-maximal lethality (LD_50_) applying 100 pg/mL of the toxin [80] that corresponds very well to the CD_50_ value of 1.31 × 10^2^ pg/mL of Stx1a (=131 pg/mL) determined by us for pHRPTEpiCs (see Figure 9). This high degree of alignment indicates an unexpected high level of reproducibility regarding the sensitivity of primary human epithelial cells derived from renal tubules of different sources toward Stx1a. In contrast to Stx1(a), subtype Stx2(a) exhibited much lower cytotoxic activity toward human renal tubular epithelial cells with an LD_50_ dose of ≈100 ng/mL [60]. A lower cytotoxicity of Stx2a versus Stx1a has been also observed by us using pHRPTEpiCs (this study), although another study reported on a reduction to 50% viability upon challenging the cells with 100 pg/mL of Stx2(a) [81]. However, the development of three-dimensional cultures of human renal tubular epithelial cells that resemble original human renal proximal tubules represents a novel in vitro model to study Stx-mediated damage and subsequent repair mechanisms after injury [62].

The SAW technology has been originally applied by us for real-time interaction analysis of Stx with Gb3Cer-spiked model membranes [82] and has been recently shown to work with influenza A virus hemagglutinins as well using neoglycolipid-loaded model membranes [83]. A substantial expansion of its use was the direct application of DRM and nonDRM preparations from sucrose gradient fractions to the functionalized surface of the biosensor. This principle has been recently for the first time employed for investigating the interaction of human-pathogenic Stx2a and swine-pathogenic Stx2e with DRM and nonDRM fractions of pHBMECs [78]. The experimental data allowed for the calculation of the dissociation constants K_D_ as a measure of the binding strength of the two Stx subtypes, i.e., the higher the value of K_D_, the lower the binding strength. SAW real-time interaction analysis of Stxs with membrane preparations of pHBMECs gave a mean K_D_ value of 77 nM and 165 nM for Stx2a and Stx2e, respectively, for DRMs, indicating a somewhat higher binding strength of Stx2a toward DRMs [78]. No interaction at all was detected for Stx2a to nonDRMs. Only a slow binding of Stx2e could be recognized that did not allow calculation of the equilibrium dissociation constant K_D_. The enrichment of Gb3Cer and Gb4Cer in DRMs and marginal content of both GSLs in nonDRMs could explain this attachment capability of the two toxins [78]. The results using DRMs and nonDRMs of pHRPTEpiCs in this study, where we compared the adhesion behavior of Stx1a and Stx2a, were different with regard to Stx2a from those obtained with the membrane preparations of pHBMECs. SAW analysis of DRMs derived from pHRPTEpiCs with Stx1a yielded a mean K_D_ of 79.5 nM for Stx1a, which is de facto identical to that observed for the interaction of Stx2a with DRMs of pHBMECs. Due to the very limited biological material of the DRMs from the primary tubular epithelial cells, combined fractions of DRM F1 to DRM F3 were applied in case of replicate 2 for the SAW investigations shown in Figure 8. In case of replicate 1, membrane material from DRM fractions F2 and F3 were combined for determining the equilibrium dissociation constant K_D_ from SAW measurements shown in Figure 9. Importantly, despite this slight difference in membrane preparations, the binding and association curves of the two replicates were almost identical. However, Stx2a exhibited only weak adhesion strength toward DRMs and nonDRMs of pHRPTEpiCs, allowing no calculation of the K_D_ constant. This lower attachment correlates with the lesser cytotoxic activity (factor 12.7) of Stx2a versus Stx1a. On the other hand, the observed difference is somewhat surprising due to the preponderance of the major Stx receptor Gb3Cer in DRMs of pHRPTEpiCs and calls for further in-depth investigation in the future.

## 4. Materials and Methods

All employed materials and methods are well established and have been described in detail in previous publications. Therefore, brief descriptions are provided together with the appropriate citations.

### 4.1. Cell Cultivation of pHRPTEpiCs

Primary human renal proximal tubular epithelial cells (pHRPTEpiCs) were purchased from ScienCell^TM^ (Carlsbad, CA, USA; Cat. No. 4100). The lower case “p” stands for “primary” to mark these kidney epithelial cells as descendants from a healthy human organ. Upon receipt of cells derived from the 1st passage, a master bank of pHRPTEpiCs of the 4th passage was established. Cryopreserved cell aliquots were stored in the gas phase over liquid nitrogen. On demand, the cells were thawed and propagated at 37 °C in a humidified atmosphere containing 5% CO_2_ using special ScienCell^TM^ epithelial cell medium (EpiCM, Cat. No. 4101) supplemented with 2% fetal bovine serum (FBS, Cat. No. 0010) and 1% epithelial cell growth supplement (EpiCGS, Cat. No. 4152) without antibiotics. At approximate 80% of confluence, the cells were trypsinized with 0.25% Trypsin-EDTA (Lonza, Verviers, Belgium; cat. CC-5012) and passaged following standard protocols [84,85]. For cell mass production, 5 × 10^6^ cell equivalents of the master bank were thawed and propagated under microscopic control in 175 cm^2^ tissue culture flasks (Greiner Bio-One, Frickenhausen, Germany) employing an Axiovert 40C microscope (Carl Zeiss AG, Oberkochen, Germany). The cell morphology was recorded with a digital camera (Canon PowerShot G10, Canon, Tokyo, Japan) and documented with AxioVison 4.8 (Carl Zeiss AG, Oberkochen, Germany). Images were processed with Adobe Photoshop software (Adobe Systems, San Jose, CA, USA). Vero-B4 cells served as the reference cell line, which was obtained from the German Collection of Microorganisms and Cell Cultures (DSMZ, Braunschweig, Germany; DSMZ no. ACC 33). Vero-B4 cells were grown in serum-free OptiPRO^TM^ SFM medium (Gibco Life Technologies Corporation, Paisley, UK; catalogue no. 12309-019) with 4 mM L-glutamine supplement and passaged by trypsinization as a matter of routine.

### 4.2. Stx Challenge and Cell Viability Assay of pHRPTEpiCs

The survival capability of pHRPTEpiCs upon Stx challenge was probed with the crystal violet assay, as previously described [71,76,82,84,86]. Briefly, cells were distributed in 100 µL volumes, each containing 4 × 10^3^ cells, to 96-well tissue culture plates (Corning Inc., Corning, NY, USA) and allowed to adhere for 24 h (37 °C, 5% CO_2_). Then, the cells were treated with increasing concentrations of affinity-purified Stx1a or Stx2a [71,82] from 1 fg/mL up to 1 µg/mL in a final cell culture volume of 200 µL, whereby cell culture medium without toxin was the 100% viability control. The cell supernatant was withdrawn after this treatment, and remnant cells were fixed with formalin. Crystal violet staining and quantitative densitometry were done as described in previous publications [71,76,82,84,86]. The obtained data correspond to means ± standard deviations (SD) of 6-fold determinations of 4 biological replicates and are portrayed as percentages related to control cells without toxin treatment consistent with a viability of 100%. The 50% cytotoxic dose (CD_50_) was defined as the Stx concentration that exerted cell death of 50% of the cells. 

### 4.3. Making of DRM and nonDRM Fractions

We followed the original description of Brown and Rose for the preparation of DRM and nonDRM fractions, which were isolated from sucrose density gradients after ultracentrifugation [87,88], and modified the protocol to a minor degree as previously reported [74,76,78,85,89]. Briefly, after disruption of the cell layers with the appropriate cell lysis buffer, the cell debris was separated by smooth centrifugation (400× *g*). The supernatant, which contains the cell membranes, was submitted to short-time ultracentrifugation (150,000× *g*). The resulting sediment was taken up in 1% Triton X-100 buffer and thoroughly mixed with an equal volume of 85% sucrose. This 42.5% sucrose solution was subsequently overlayed step by step with solutions of 30% and 5% sucrose followed by ultracentrifugation (200,000× g). Afterwards, three DRM top fractions (F1 to F3) and five nonDRM fractions were obtained, whereby the latter were further subdivided into three lower nonDRM fractions (F4 to F6, intermediate) and the two lowest nonDRM fractions (F7 to F8, bottom). These eight samples, each corresponding to a volume of 1.5 mL, were taken stepwise from top to bottom and analyzed for their lipid composition (see next paragraphs). 

### 4.4. Extraction of Lipids and Purification of GSLs from Total Cells

Lipid extraction was performed with two independently produced approaches of in vitro grown pHRPTEpiCs following previously published instructions [13,71,76,85]. Methanol extraction was continued by treatment of the cell slurry with chloroform/methanol (1/2, *v/v*), chloroform/methanol (1/1, *v/v*), and chloroform/methanol (2/1, *v/v*). The alkali-labile triglycerides and glycerophospholipids were saponified using 1 M methanolic NaOH, followed by careful dropwise neutralization of the sample with 10 M HCl. After dialysis against deionized water und lyophilization, the dry extract was suspended in chloroform/methanol/water (30/60/8, *v*/*v/v*), and neutral GSLs were isolated by anion-exchange chromatography on column packed with DEAE-Sepharose CL-6B (GE Healthcare, Munich, Germany) as described in earlier times [90]. Finally, purified neutral GSLs were dissolved in chloroform/methanol (2/1, *v/v*) and stored at −20 °C until use.

### 4.5. Sampling of Phospholipids and GSLs in DRM and nonDRM Fractions

A short explanation of the used methods, which have been described previously [71,74,76], is given. Shortly, sucrose was removed from the gradient fractions F1 to F8 (each 1.5 mL volume, see above) by two-day dialysis. Volumes of 1.4 mL of each fraction were submitted to lyophilization. For ensuing phospholipid analysis, the dry samples were taken up in chloroform/methanol (2/1, *v*/*v*) and dissolved in a defined volume matched to 1 × 10^5^ cells/µL. Remnant 0.1 mL aliquots of the dialyzed gradient fractions, scheduled for GSL and cholesterol analysis, were lyophilized and incubated in 1 M methanolic NaOH for 1 h at 37 °C to saponify alkali-labile triglycerides and glycerophospholipids. Afterwards, the samples were neutralized with 10 M HCl, followed by dialysis and lyophilization. Finally, the samples were dissolved in chloroform/methanol (2/1, *v*/*v*) in a concentration corresponding to 1 × 10^5^ cells/µL.

### 4.6. Anti-GSL Antibodies, Affinity-Purified Stx1a and Stx2a, Secondary Antibodies, and Lipid References

Polyclonal chicken IgY anti-Gb3Cer and anti-Gb4Cer antibodies were used for overlay detection of TLC-separated total GSLs derived from pHRPTEpiCs and sucrose gradients fractions (see above). Details can be drawn from numerous previous publications [13,17,77] and a very recent dissemination that contains protocols explaining all the laboratory details and trickery practical handling of GSLs and their detection with antibodies and lectins exemplified by various Stx subtypes [91].

The Stx subtypes Stx1a and Stx2a (formerly designated as Stx1 and Stx2, now renamed as Stx1a and Stx2a compliant with the changed nomenclature of Scheutz and co-workers [92] were affinity-purified by means of Gb3-functionalized magnetic beads. The sources for Stx1a and Stx2a were sterile filtrated supernatants from bacterial liquid cultures of *Escherichia coli* wild-type strains 2074/97 (serotype O145:H−) and 03-06016 (serotype O111:H-), respectively [82]. Both affinity-purified subtypes have been previously characterized with regard to GSL binding specificity and cytotoxic action to a number of differing cell types [17,47,71,78]. Murine monoclonal IgG antibodies against Stx1 and Stx2 produced with hybridoma clones VT109/4-E9 and VT 135/6-B9, respectively, were purchased from SIFIN GmbH (Berlin, Germany). Polyclonal affinity-purified rabbit anti-chicken IgY and goat anti-mouse IgG antibody, both linked with alkaline phosphatase (AP), were used as secondary antibodies (Dianova, Hamburg, Germany, Code 303-055-033 and Code 115-055-003, respectively).

A mixture of neutral GSLs, prepared from human erythrocytes and containing the globo-series GSLs Gb3Cer (Galα1-4Galβ1-4Glcβ1-1Cer) and Gb4Cer (GalNAcβ1-3Galα1-4Galβ1-4Glcβ1-1Cer), was employed as reference and positive control in the TLC overlay assays [89,93,94,95]. Cholesterol (Sigma Aldrich, Steinheim, Germany; cat. no. C8667) was employed as reference for TLC analysis of the DRM and nonDRM fractions derived from sucrose gradients according to previous publications [76,77,89]. 

### 4.7. High-Performance Thin-Layer Chromatography, Staining of Lipids, and Overlay Assay 

The separation of total neutral GSLs isolated from pHRPTEpiCs as well as the GSL preparations derived from DRM and nonDRM fractions was performed with high-performance thin-layer chromatography (TLC) plates glass-backed and coated with silica gel 60 (HPTLC plates, size 10 cm × 10 cm, thickness 0.2 mm, no. 1.05633.0001; Merck, Darmstadt, Germany). The samples were applied to the silica gel layer on the plate surface using a semi-automatic sample applicator (Linomat 5, CAMAG, Muttenz, Switzerland). Neutral GSLs were chromatographed in the solvent composed of chloroform/methanol/water (120/70/17), *v*/*v*/*v*, while chloroform/acetone (96/4, *v*/*v*) served as the solvent for the detection of cholesterol. Orcinol was used for staining of GSLs, and cholesterol was detected with manganese(II)chloride after TLC separation as described in detail in previous publications [78,86,96]. 

TLC overlay detection was conducted with polyclonal chicken anti-Gb3Cer and anti-Gb4Cer antibodies as well as with affinity-purified Stx1a and Stx2a together with the appropriate anti-Stx1 and anti-Stx2 antibody, respectively (see above) following previously published protocols [42,76,97]. Briefly, the impregnated silica gel layer was overlayed after TLC separation of the analytes with primary anti-GSL antibodies (1:2000 diluted) or solutions with affinity-purified Stx1a or Stx2a (0.33 µg/mL each). GSL-bound anti-Gb3Cer and anti-Gb4Cer antibodies were detected with 1:2000 diluted AP-linked anti-chicken IgY antibody. Binding of Stx1a and Stx2a was evaluated with anti-Stx1 and anti-Stx2 antibody (each 1:2000 diluted) and the appropriate AP-conjugated goat anti-mouse IgG antibody (1:2000 dilution). 5-bromo-4-chloro-3-indolyl phosphate *p*-toluidine salt (BCIP, Roth, Karlsruhe, Germany) served as the substrate for color development of recognized GSLs used as 0.05% (*w*/*v*) in glycine solution (pH 10.4) generating a blue precipitate at positions of bound anti-GSL antibodies or Stxs on the TLC plate.

### 4.8. Mass Spectrometric Analysis of GSLs and Phospholipids

The structures of GSLs and phospholipids were analyzed by means of nano electrospray ionization mass spectrometry (nanoESI MS) using a SYNAPT G2-S mass spectrometer (Waters, Manchester, UK) endowed with a Z-spray as previously described [13,14,17,71]. In short, GSL were structurally characterized in positive ion mode with the following source settings: temperature 80 °C, capillary voltage 0.8 kV, sampling cone voltage 20 V, and offset voltage 50 V. Verification of MS^1^ postulated GSL and phospholipid structures were verified by low-energy collision-induced dissociation (CID) MS^2^ experiments. For this purpose, analyte precursor ions were selected in the quadrupole analyzer and separated by ion mobility under the following conditions: wave velocity 700–800 m/s, wave height 40 V, nitrogen gas flow rate 90 mL/min, and helium gas flow rate 180 mL/min. Ion fragmentation was achieved in the transfer cell using collision energies of 70 to 100 eV (E_lab_). The nomenclature established by Domon and Costello served for denomination of MS^2^-derived GSL fragment ions [98,99]. 

### 4.9. Surface Acoustic Wave Technology and Biomolecular Interaction Analysis in Real Time

The previously established methodology for real-time biomolecular interaction analysis of Stxs with Gb3Cer-endowed model membranes [82] was applied to perform direct interaction analysis of affinity-purified Stx1a and Stx2a with DRM and nonDRM fractions prepared from pHRPTEpiCs. Briefly, small unilamellar vesicles were produced with pooled DRM top fractions F1 to F3 and the nonDRM bottom fraction F7, respectively, as previously described in detail [82] introducing some minor modifications. Shortly, small unilamellar vesicles were produced by the extrusion of multilamellar vesicles through a 100 nm pore-sized polycarbonate membrane (Whatman^®^ Nucleopore^TM^ Track-Etched Membranes, GE Healthcare, Maidstone, UK) by means of a mini-extruder (Avanti Polar Lipids Inc., Alabaster, AL, USA). The surface acoustic wave (SAW) device SAM^®^5 blue (SAW Instruments GmbH, Bonn, Germany) was used for label-free biomolecular interaction analysis in real time. The aureate chip surface was functionalized with 11-mercaptoundecanoic acid and then loaded with the lipid vesicles using concentrations of 1 mg/mL. Stx1a and Stx2a subtypes were taken up in phosphate-buffered saline (PBS) with 5 mM MgCl_2_ and injected with rising concentrations of 20 nM up to 250 nM. After measurement, remnant Stx was eluted from the biomembrane with 0.5 M melibiose (Galα1-6Glc; melibiose monohydrate, cat. no. 4223.3, Carl Roth GmbH+Co KG, Karlsruhe, Germany) dissolved in PBS with 5 mM MgCl_2_. The association and dissociation rate constants *k_ass_* and *k_diss_*, deduced from the calculated values of *k_obs_* (“obs” stands for “observed”) and the concentration *c* of the Stx ligand, were mathematically defined from the slope of the linear regression function according to the equation *k_obs_* = *k_ass_* × *c* + *k_diss_*. Calculation of the equilibrium dissociation constant *K_D_* was done by means of the quotient formation *K_D_* = *k_diss_*/*k_ass_* following the previously published instructions [82]. 

## 5. Conclusions

The present study suggests that renal proximal tubular epithelial cells might play a substantial role with regard to Stx1a-mediated kidney injury during the development of HUS, whereas the cell-damaging role of Stx2a toward proximal tubules remains somewhat unclear. However, our study adds a further piece to the puzzle of events that accumulate in the sophisticated process of kidney failure during the course of HUS. 

## Figures and Tables

**Table 1:** Main GSLs and SM of pHRPTEpiCs characterized by mass spectrometry in combination with Stx and TLC overlay immunodetection ^1^.

Compound ^2^	Fatty Acid	Formula	*m/z*_exp_^3^	*m/z*_calc_^3^
SM	C16:0	C_39_H_79_N_2_O_6_PNa	725.56	725.5573
SM	C22:0	C_45_H_91_N_2_O_6_PNa	809.65	809.6512
SM	C24:1	C_47_H_93_N_2_O_6_PNa	835.66	635.6669
SM	C24:0	C_47_H_95_N_2_O_6_PNa	837.67	837.6825
MHC	C16:0	C_40_H_77_NO_8_Na	722.55	722.5547
MHC	C22:0	C_46_H_89_NO_8_Na	806.65	806.6486
MHC	C24:1	C_48_H_91_NO_8_Na	832.66	832.6642
MHC	C24:0	C_48_H_93_NO_8_Na	834.68	834.6799
Lc2Cer	C16:0	C_46_H_87_NO_13_Na	884.61	884.6075
Lc2Cer	C22:0	C_52_H_99_NO_13_Na	968.69	968.7014
Lc2Cer	C24:1	C_54_H_101_NO_13_Na	994.71	994.7171
Lc2Cer	C24:0	C_54_H_103_NO_13_Na	996.73	996.7327
Gb3Cer	C16:0	C_52_H_97_NO_18_Na	1046.66	1046.6603
Gb3Cer	C22:0	C_58_H_109_NO_18_Na	1130.75	1130.7542
Gb3Cer	C24:1	C_60_H_111_NO_18_Na	1156.76	1156.7699
Gb3Cer	C24:0	C_60_H_113_NO_18_Na	1158.78	1158.7855
Gb4Cer	C16:0	C_60_H_110_N_2_O_23_Na	1249.74	1249.7397
Gb4Cer	C22:0	C_66_H_122_N_2_O_23_Na	1333.83	1333.8336
Gb4Cer	C24:1	C_68_H_124_N_2_O_23_Na	1359.85	1359.8493
Gb4Cer	C24:0	C_68_H_126_N_2_O_23_Na	1361.86	1361.8649
Gb5Cer	C16:0	C_66_H_120_N_2_O_28_Na	1411.78	1411.7925
Gb5Cer	C22:0	C_72_H_132_N_2_O_28_Na	1495.89	1495.8864
Gb5Cer	C24:1	C_74_H_134_N_2_O_28_Na	1521.90	1521.9021
Gb5Cer	C24:0	C_74_H_136_N_2_O_28_Na	1523.91	1523.9177

^1^ GSLs and SM derived from replicate 2 of pHRPTEpiCs were analyzed; the oligosaccharides of the Gb3Cer and Gb4Cer species were determined with anti-Gb3Cer and anti-Gb4Cer antibodies, respectively, and confirmed with Stx1a and Stx2a (see Figure 1B,C, respectively); all detected GSLs and SM carried sphingosine (d18:1) in their respective ceramide moieties ^2^ GSLs and SM were detected in the positive ion mode as monosodiated [M+Na]^+^ species; examples of MS^2^ spectra are shown for Gb3Cer (d18:1, C22:0), Gb4Cer (d18:1, C16:0), and proposed Gb5Cer (d18:1, C22:0) in Figures S2–S4, respectively, in the Supplementary Materials; ^3^ exp, experimental; calc, calculated.

## Data Availability

Data produced throughout the study are available from the corresponding author upon reasonable request.

## Supplementary Materials

The following are available online at https://www.mdpi.com/article/10.3390/toxins13080529/s1, Figure S1: Light microscopy micrographs of pHRPTEpiCs during passage 3 (P3), passage 8 (P8), and passage 13 (P13) at approximate 30% confluence, Figure S2: MS2 spectrum of Gb3Cer (d18:1, C22:0) obtained from pHRPTEpiCs (A) and corresponding fragmentation scheme (B), Figure S3: MS2 spectrum of Gb4Cer (d18:1, C16:0) obtained from pHRPTEpiCs (A) and corresponding fragmentation scheme (B), Figure S4: MS2 spectrum of Gb5Cer obtained from pHRPTEpiCs (d18:1, C22:0) (A) and corresponding fragmentation scheme (B), Figure S5: SAW real-time interaction sensorgrams gained for binding of Stx1a toward DRM (A) and nonDRM fractions (B) prepared from replicate 1 of pHRPTEpiCs, Figure S6: SAW real-time interaction sensorgrams gained for binding of Stx2a toward DRM fractions prepared from replicate 1 of pHRPTEpiCs, Table S1: Relative distribution of Gb3Cer, Gb4Cer, and cholesterol in sucrose gradient fractions obtained from pHRPTEpiCs, Table S2: Calculated association kass and dissociation kdiss rate constants and equilibrium dissociation constant KD for Stx1a using DRMs of pHRPTEpiCs.
