# Supplementary material for: Primary Human Renal Proximal Tubular Epithelial Cells (pHRPTEpiCs): Shiga Toxin (Stx) Glycosphingolipid Receptors, Stx Susceptibility, and Interaction with Membrane Microdomains
Source: Toxins (Basel). 2021 Jul 28;13(8):529. doi: 10.3390/toxins13080529 (PMC8402424; doi:10.3390/toxins13080529)
Supplement: Supplementary file 1 [file toxins-13-00529-s001.zip › toxins-1246918-supplementary.pdf]

Supplementary Materials: Primary Human Renal Proximal Tubular Epithelial Cells (pHRPTEpiCs): Shiga Toxin (Stx) Glycosphingolipid Receptors, Stx Susceptibility and Interaction with Membrane Microdomains

Johanna Detzner, Anna-Lena Klein, Gottfried Pohlentz, Elisabeth Krojnewski, Hans-Ulrich Humpf, Alexander Mellmann, Helge Karch and Johannes Müthing

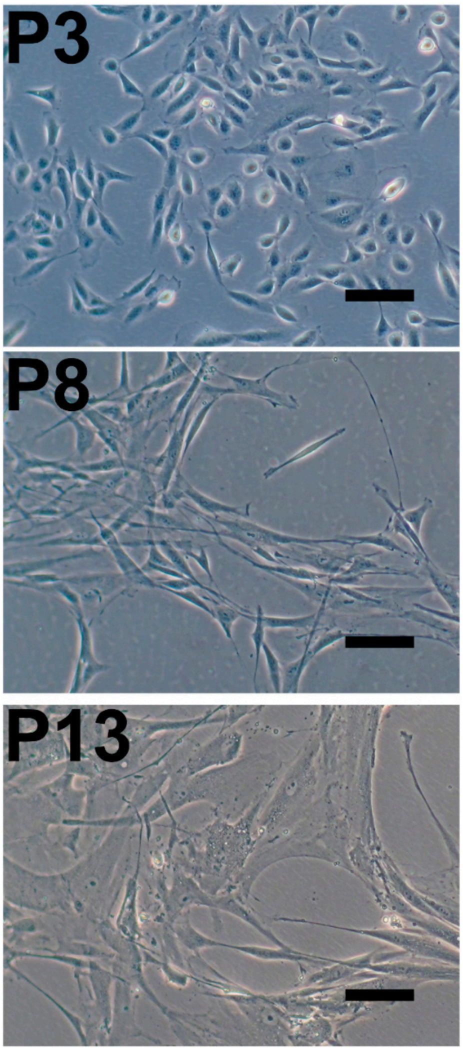

Figure S1. Light microscopy micrographs of pHRPTEpiCs during passage 3 (**P3**), passage 8 (**P8**), and passage 13 (**P13**) at approximate 30% confluence. Original magnification ×10. Bar: 100 µm.

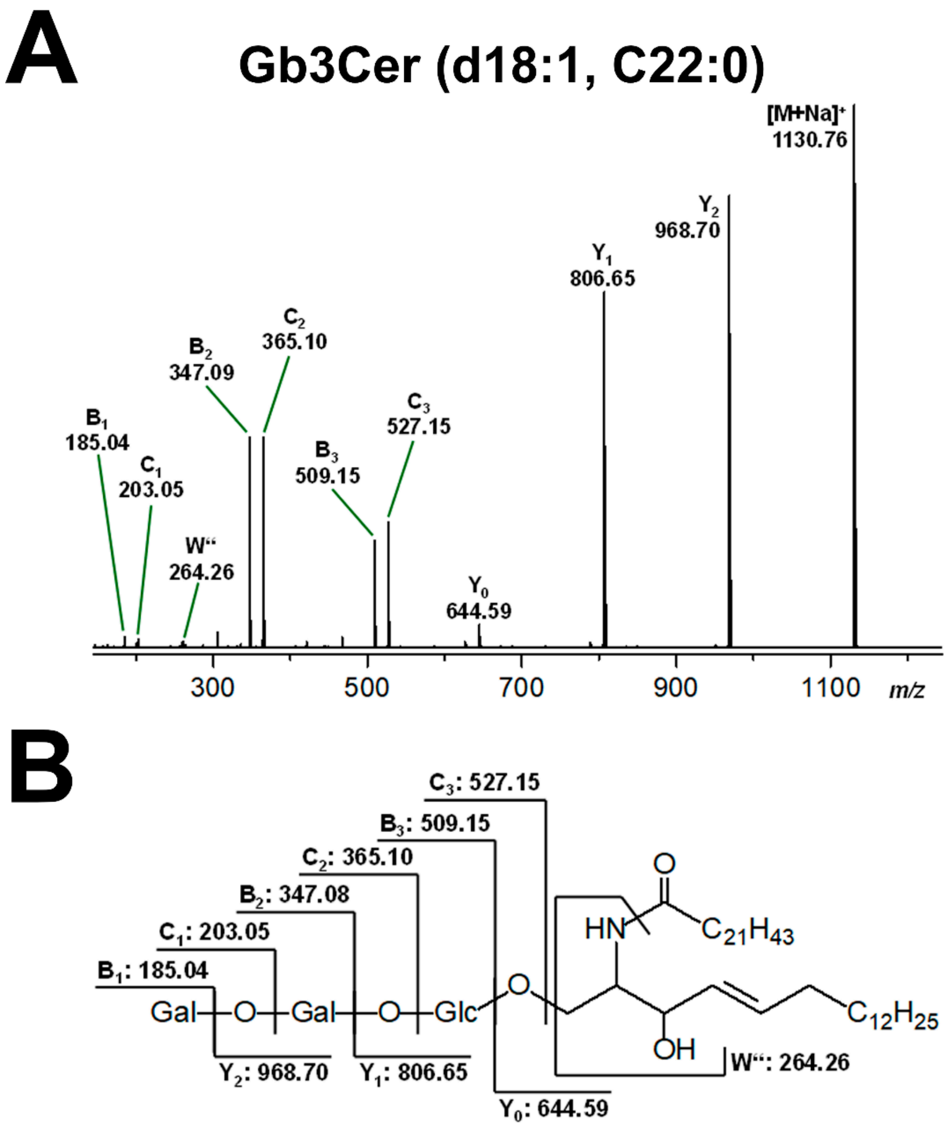

Figure S2. MS^2 spectrum of Gb3Cer (d18:1, C22:0) (**A**) and corresponding fragmentation scheme (**B**) obtained from pHRPTEpiCs.

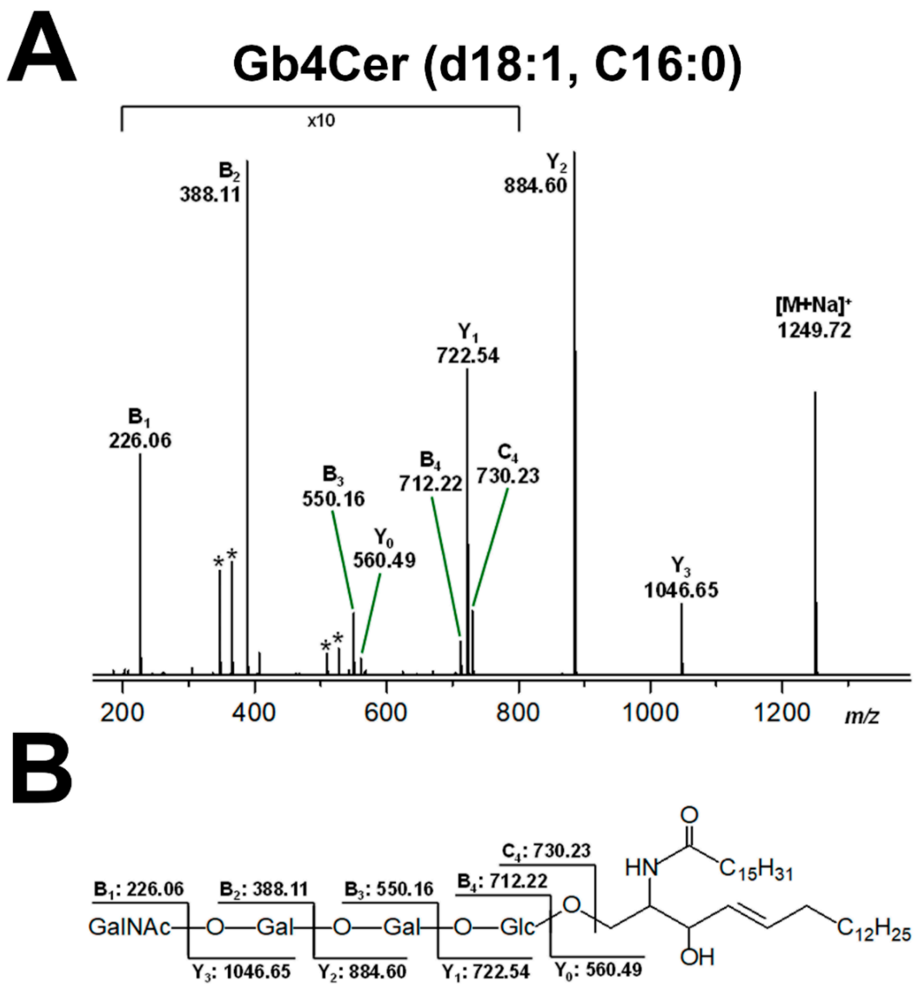

Figure S3. MS[2] spectrum of Gb4Cer (d18:1, C16:0) (**A**) and corresponding fragmentation scheme (**B**) obtained from pHRPTEpiCs.

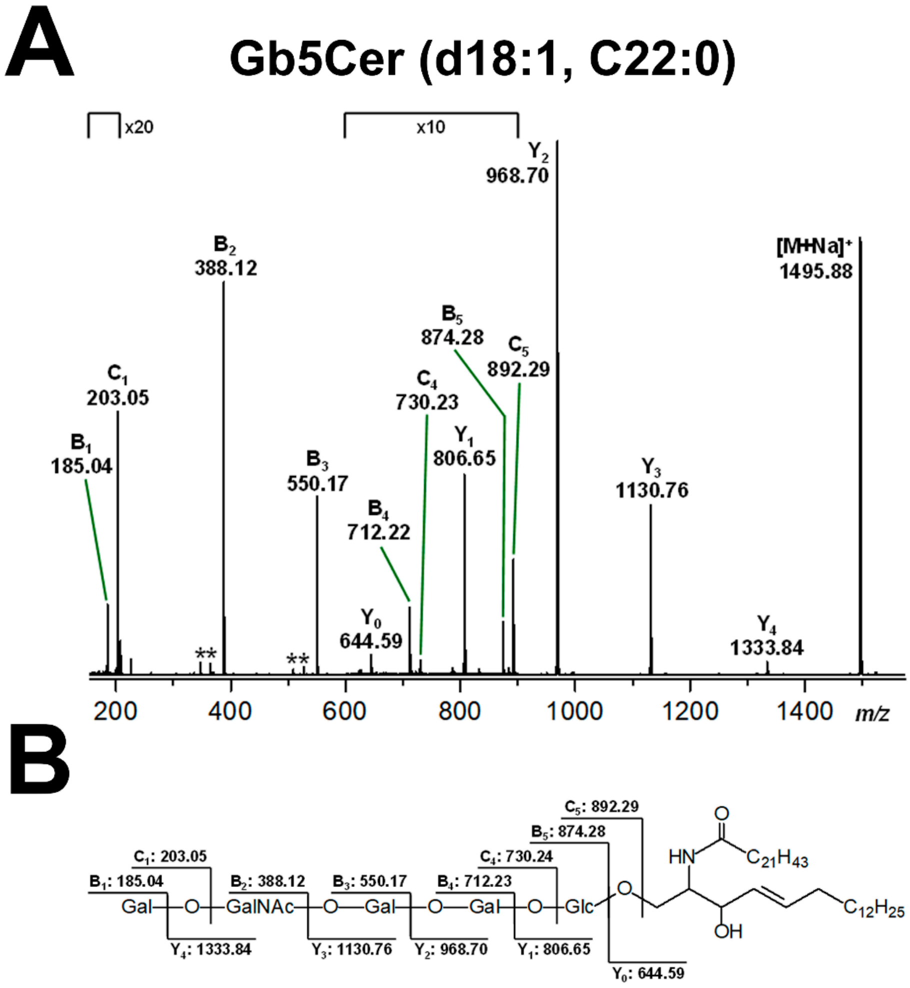

Figure S4. MS² spectrum of Gb5Cer (d18:1, C22:0) (**A**) and corresponding fragmentation scheme (**B**) obtained from pHRPTEpiCs.

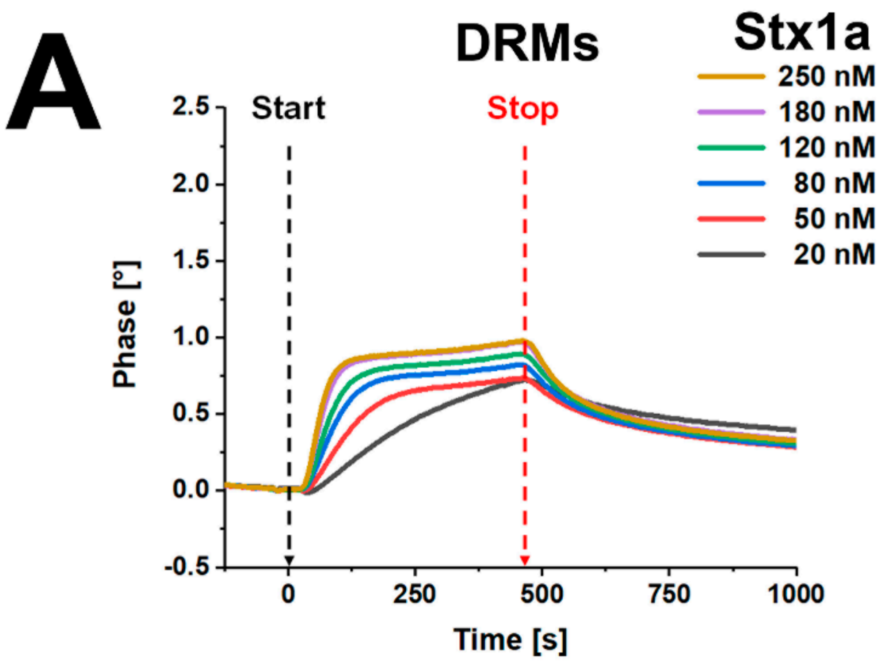

Figure S5. SAW real-time interaction sensorgrams gained for binding of Stx1a towards DRM (**A**) and nonDRM fractions (**B**) prepared from replicate 1 of pHRPTEpiCs.

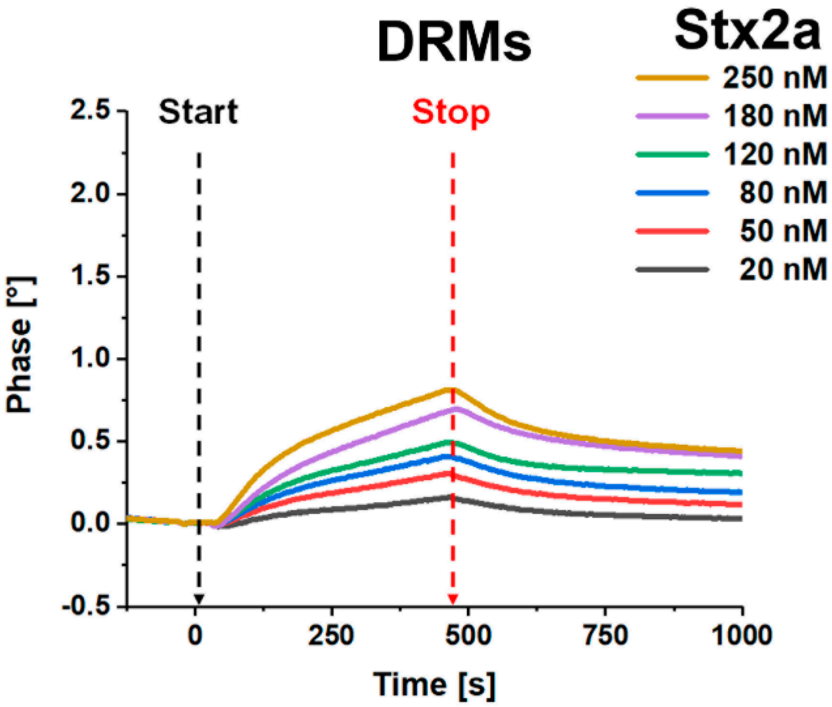

Figure S6. SAW real-time interaction sensorgrams gained for binding of Stx2a towards DRM fractions prepared from replicate 1 of pHRPTEpiCs.

Table S1. Relative distribution of Gb3Cer, Gb4Cer, and cholesterol in sucrose gradient fractions obtained from pHRPTEpiCs.

Gb3Cer

Fraction	Replicate 1 (%)		Replicate 2 (%)	
F1 DRM (top)	0.0		14.8	
F2 DRM (top)	49.6		48.9	
F3 DRM (top)	16.4	Σ66.0 (F1–F3)	14.1	Σ77.8 (F1–F3)
F4 nonDRM (intermediate)	7.7		8.1	
F5 nonDRM (intermediate)	8.9		5.4	
F6 nonDRM (intermediate)	8.4	Σ25.0 (F4–F6)	0.0	Σ13.5 (F4–F6)
F7 nonDRM (bottom)	7.0		7.7	
F8 nonDRM (bottom)	2.0	Σ9.0 (F7–F8)	1.0	Σ8.7 (F7–F8)
		Σ100.0 (F1–F8)		Σ100.0 (F1–F8)

Gb4Cer

Fraction	Replicate 1 (%)		Replicate 2 (%)	
F1 DRM (top)	0.0		10.1	
F2 DRM (top)	64.9		59.1	
F3 DRM (top)	15.9	Σ80.8 (F1–F3)	11.7	Σ80.9 (F1–F3)
F4 nonDRM (intermediate)	5.4		5.9	
F5 nonDRM (intermediate)	5.0		2.1	
F6 nonDRM (intermediate)	3.8	Σ14.2 (F4–F6)	0.0	Σ8.0 (F4–F6)
F7 nonDRM (bottom)	5.0		9.8	
F8 nonDRM (bottom)	0.0	Σ5.0 (F7–F8)	1.3	Σ11.1 (F7–F8)
		Σ100.0 (F1–F8)		Σ100.0 (F1–F8)

Cholesterol

Fraction	Replicate 1 [%]		Replicate 2 [%]	
F1 DRM (top)	0.0		4.8	
F2 DRM (top)	57.4		47.5	
F3 DRM (top)	13.3	Σ70.7 (F1–F3)	14.9	Σ67.2 (F1–F3)
F4 nonDRM (intermediate)	6.1		9.3	
F5 nonDRM (intermediate)	9.0		7.8	
F6 nonDRM (intermediate)	5.3	Σ20.4 (F4–F6)	0.0	Σ17.1 (F4–F6)
F7 nonDRM (bottom)	7.5		14.1	
F8 nonDRM (bottom)	1.4	Σ8.9 (F7–F8)	1.6	Σ15.7 (F7–F8)
		Σ100.0 (F1–F8)		Σ100.0 (F1–F8)

Table S2. Calculated association k_{ass} and dissociation k_{diss} rate constants and equilibrium dissociation constant K_D for Stx1a using DRMs of pHRPTEpiCs[a].

Sensor channel	k_{ass} (nM^{-1} s^{-1})[b]	k_{diss} (s^{-1})[b]	K_D (nM)[b]
1	1.0×10^{-4}	8.8×10^{-3}	88.3
2	1.0×10^{-4}	8.2×10^{-3}	81.8
3	1.0×10^{-4}	7.4×10^{-3}	73.9
4	0.9×10^{-4}	6.9×10^{-3}	76.2
5	0.8×10^{-4}	6.2×10^{-3}	77.5
Mean value	0.9×10^{-4}	7.5×10^{-3}	79.5
SD[c]	8.9×10^{-6}	1.0×10^{-3}	5.7

[a] Constants were determined by real-time interaction analysis of Stx1a with sensor surface-coated pooled DRM fractions F1-F3 prepared from replicate 1 of pHRPTEpiCs; [b] k_{ass}, k_{diss} and K_D values were obtained for Stx1a and correspond to sensorgrams of Figure 12; [c] SD, standard deviation.
